# Case of Inflammation of the Spinal Cord

**Published:** 1859-02

**Authors:** Jno. C. K. Crooks

**Affiliations:** Honeoye, N.Y.


ART. II.—*Case of Inflammation of the Spinal Cord.* By JNO. C. K. CROOKS, M. D., Honeoye, N. Y.

During the evening of September 29th, 1857, I was called to visit a young man, aged 17, who, I was informed by the messenger, was suffering from obstinate constipation, difficult breathing, and sundry other troubles, of which he could give but a vague account. Ascertaining that he had been ill for several days, and that he had had the professional services of a very worthy retired practitioner, who resided in his neighborhood, I resolved to postpone my visit till the following morning, and informed the messenger to that effect. Accordingly, in the morning, I called at an early hour, and found, greatly to my surprise, that the young man was laboring under a partial loss of sensibility, and almost complete paralysis of the whole body, below the origin of the phrenic nerve.

A history of the case revealed the following: Five days previous to my visit, after having labored hard upon the farm, pitching sheaves from a stack to a threshing machine, he began to suffer from a dull, heavy pain in the nape of the neck, and weariness of the upper extremities. At first, it was not severe, but becoming worse, and accompanied with a sense of constriction of the chest, his parents became alarmed, and the physician referred to was called in. He considered the whole trouble the effects of "cold and over exertion." Rest was strictly enjoined, and a gentle cathartic administered, to be followed with a Dover's powder, if the young man was unable to sleep from the difficulty of breathing. One or two repetitions of the cathartic procured an evacuation of the bowels, but no relief followed. Laxatives were then administered, but without effect, and small doses of pulv. Dov. and calomel, at short intervals. This constituted the treatment, with but little variation, up to the time that I was called, the symptoms of grave disease of the spinal cord becoming more and more marked.

The constriction of the chest, or sense of suffocation, was first observed; then came a loss of power in the right arm, then in the right leg, then in the left arm, but to a less degree, and so on to the left inferior extremity, at the same time the sense of suffocation becoming greatly increased. In this condition I found him. He looked pale and anxious; the tongue was dry, with a brown fur in the centre; pulse 110, full, but quite irregular; the right leg was in a state of complete paralysis; the left could be rotated, slightly flexed and extended, but with a great deal of difficulty, and it seemed to exhaust the patient. The motions of the right arm were all arrested except the power to flex and extend the fingers, while the left was much stronger than the corresponding inferior extremity. He could void his urine, but with much trouble, being accompanied with exhaustion, as in exercising, or *trying* to exercise the extremities. In evacuating the bowels, he experienced still more difficulty, as the exertion greatly prostrated him, and occasioned anxious and labored respiration. His respiration was *entirely* abdominal, there being not the least perceptible motion to the walls of the chest. They were completely fixed — *motionless.* At times, there were severe colicy pains in different parts of the abdomen, and occasional frontal cephalalgia.

Upon examining the spine, I found the lower cervical and upper dorsal regions extremely sensitive to pressure, while between two of the upper dorsal vertebræ, there was an abrupt angular displacement — a lateral curvature. At, and above the curvature, the sensitiveness was most marked. I considered the curvature an old difficulty, and a predisposing cause for the present trouble, which was, evidently, acute inflammation of the spinal cord.

As calomel had been given in considerable quantity, I ordered a cathartic of castor oil and turpentine, to be repeated in four hours, and then, if no operation was procured, to administer stimulating enemata. I depleted topically, by scarifying and cupping along the sides of the spine, and followed the bleeding by a pretty free application of strong iodide of mercury oint. His feet were placed in a mustard pediluvium, and after removal, were enclosed as well as his legs, in mustard plasters. He was then placed in a comfortable bed, in a semi-recumbent position, to relieve, as much as possible, all embarrassment to the motions of the diaphragm; and, as the weather was fine, a free access to the external air given.

*Oct. 1st.* Patient much as upon the day previous; pulse 108, irregular; no evacuation of the bowels.

Ordered croton oil, to be followed by olium ricini and turpentine, and enemas of strong salt water, in large quantities, often repeated.

The respiration was still difficult, particularly toward evening.

Renewed the iodide of mercury oint.; extremities kept warm.

*Oct. 2d.* Slight change for the better; pulse 100, irregular; not quite as full; bowels had operated freely. The spirits of the patient had improved somewhat, as the difficulty of breathing was lessened; spine still very sensitive to pressure. As the gums were untouched by the previous use of mercury, I ordered calomel in small doses, frequently repeated, and unguarded by opium, as the bowels were strongly inclined to constipation; urine voided with less difficulty.

*Oct. 3d.* Having had an obstetrical case in charge, I did not see my patient till late in the evening. The weather was heavy, and a good deal of rain had been falling during the day. I found the young man apparently much worse; respiration anxious; pain in the bowels; pulse 110 again, and irregular; extremities cold, &c.

I ordered the mustard pediluvium repeated; administered stimulating enemata, which, together with encouraging words, soon restored the spirits somewhat, and quieted, in a degree, the labored respiration; gums remained untouched.

Continued cal., and re-applied the ointment to the spine.

*Oct. 4th.* Patient much improved; pulse 90, but irregular still; tongue becoming moist, and disposed to clean; *gums tender;* had had one or two dejections since last visit; respiration less labored; no improvement in the paralysis of the extremities; spine less tender.

From this time the young man slowly improved for several days; tenderness over the spine became less and less marked; respiration was better; tongue cleaned; bowels moved without much difficulty, and he began to have considerable appetite.

Suddenly the action of the heart began to falter, and its pulsations rapidly ran down to thirty-six per minute. Although I feared that disorganization of the spinal cord was going on, softening, induration, or suppuration, I determined to put him upon strichnine, and accordingly did so, beginning cautiously, and increasing as rapidly as the circumstances would allow.

For several days there seemed to be but slight amendment, the pulsations of the heart being about forty in the evening, and forty-five in the morning, after the rest of the night. Then they began slowly to increase, till they arrived at at sixty-five per minute, where they remained stationary for weeks. As the heart recovered its vigor of action, the respiration improved still more, as also the strength of the extremities, but to a much less degree. His appetite now became excellent, and the accumulation of adipose tissue was quite remarkable in its rapidity.

After the lapse of two or three months from the beginning of the attack, he was able to stand erect, after being assisted upon his feet, and by being supported, he could slide his feet along the smooth floor, and make a little progress in walking; general sensation had greatly improved.

I now gave his parents, who were intelligent pecple, general directions as to treatment, regimen, &c.

Ordered the continuance of counter irritants to the spine, the extremities kept warm, regular exercise in the open air, when the weather would admit, cold sponge bath, with frictions, continuance of strichnine, alternating with the use of electricity, &c.

I now heard from my patient occasionally, and learned that he was steadily improving, furnishing him with remedies, whenever his supply was exhausted. This occurred for three or four months, when I had no more intelligence from him, till August 14th and 28th, 1858, when I called upon him. I found that he had greatly improved in the interim, although his parents were much discouraged with the prospects of his case. He could walk quite well, with the assistance of a cane, but could not yet go up stairs, or ascend a steep hill, from the weakness of the extensor muscles of the inferior extremities. For the same reason, whenever he accidentally fell, he could not arise without assistance. His respiration was good, the muscles of the walls of the chest having recovered their vigor to a great extent. His sensation was nearly natural; his bowels were regular, and he voided urine at all times, without trouble.

Notwithstanding this improvement in the functions of the spinal cord, he looked cachectic; had lost his fat; pulse was a little too rapid; became fatigued easily; coughed slightly, and although I could detect no pulmonary difficulty, I fear that tuberculosis is impending.

I recommended a general tonic treatment, gentle counter irritation over the chest, and a continuance of strychnine, which for some time had been omitted, in *greatly increased doses.*

Illness of myself, and a long absence at the west, have prevented my seeing him again. There are several peculiarities in his case — points of interest, of which I might speak, but I have already taxed your patience at too great length. More anon.

